# Allogeneic human umbilical cord Wharton’s jelly stem cells increase several-fold the expansion of human cord blood CD34+ cells both in vitro and in vivo

**DOI:** 10.1186/s13287-020-02048-0

**Published:** 2020-12-09

**Authors:** Hao Daniel Lin, Chui-Yee Fong, Arijit Biswas, Ariff Bongso

**Affiliations:** Department of Obstetrics and Gynaecology, Yong Loo Lin School of Medicine, National University Health System, National University of Singapore, Kent Ridge, 119228 Singapore

**Keywords:** Conditioned medium, Ex vivo expansion, Human Wharton’s jelly stem cells, Glycolysis, Mitochondria, Mouse model, Oxidative phosphorylation, Umbilical cord blood CD34+ cells

## Abstract

**Background:**

The transplantation of human umbilical cord blood (UCB) CD34+ cells has been successfully used to treat hematological disorders but one major limitation has been the low cell numbers available. Mesenchymal stem cells (MSCs) lying within the bone marrow in vivo behave like a scaffold on which CD34+ cells interact and proliferate. We therefore evaluated the use of allogeneic MSCs from the human UC Wharton’s jelly (hWJSCs) as stromal support for the ex vivo expansion of CD34+ cells.

**Methods:**

We performed an in-depth evaluation of the primitiveness, migration, adhesion, maturation, mitochondrial behavior, and pathway mechanisms of this platform using conventional assays followed by the evaluation of engraftment potential of the expanded CD34+ cells in an in vivo murine model.

**Results:**

We demonstrate that hWJSCs and its conditioned medium (hWJSC-CM) support the production of significantly high fold changes of CD34+, CD34+CD133+, CD34+CD90+, CD34+ALDH+, CD34+CD45+, and CD34+CD49f+ cells after 7 days of interaction when compared to controls. In the presence of hWJSCs or hWJSC-CM, the CD34+ cells produced significantly more primitive CFU-GEMM colonies, HoxB4, and HoxA9 gene expression and lower percentages of CD34+CXCR4+ cells. There were also significantly higher N-cadherin+ cell numbers and increased cell migration in transwell migration assays. The CD34+ cells expanded with hWJSCs had significantly lower mitochondrial mass, mitochondrial membrane potential, and oxidative stress. Green Mitotracker-tagged mitochondria from CD34+ cells were observed lying within red CellTracker-tagged hWJSCs under confocal microscopy indicating mitochondrial transfer via tunneling nanotubes. CD34+ cells expanded with hWJSCs and hWJSC-CM showed significantly reduced oxidative phosphorylation (ATP6VIH and NDUFA10) and increased glycolytic (HIF-1a and HK-1) pathway-related gene expression. CD34+ cells expanded with hWJSCs for 7 days showed significant greater CD45+ cell chimerism in the bone marrow of primary and secondary irradiated mice when transplanted intravenously.

**Conclusions:**

In this report, we confirmed that allogeneic hWJSCs provide an attractive platform for the ex vivo expansion of high fold numbers of UCB CD34+ cells while keeping them primitive. Allogeneic hWJSCs are readily available in abundance from discarded UCs, can be easily frozen in cord blood banks, thawed, and then used as a platform for UCB-HSC expansion if numbers are inadequate.

## Introduction

Hematopoietic stem cell (HSC) transplantation has been used for the treatment of malignant and non-malignant hematopoietic diseases such as leukemia, lymphoma, and thalassemia. However, the aspiration of HSCs from the bone marrow is painful with the potential risk of infection and morbidity. Optimal HSC numbers are not always available and matched samples need to be sought for successful transplantation. To overcome these disadvantages, HSCs from the human umbilical cord blood (UCB) have been used for the treatment of hematopoietic diseases in children in autologous and allogeneic settings [1, 2]. The clinical benefits of UCB stem cell transplantation have been comprehensively reviewed [3]. UCB contains HSCs and hematopoietic progenitor cells (HPCs) that appear to have higher proliferation rates and immunological tolerance compared to those in the bone marrow [4]. One major hurdle associated with the use of UCB-HSCs for transplantation is the relatively low cell numbers available. This has contributed to slower engraftment and increased risk of engraftment failure [5]. It has been reported that HSC and HPC numbers in UCB were adequate for the treatment of hematopoietic diseases in children and not adults as it is been estimated that for successful engraftment at least 2.5 × 10^6^ CD34+ cells per kilogram of patient body weight is required [6, 7]. Unfortunately, a good UCB harvest from a single umbilical cord generates only a total of about 10 × 10^6^ CD34+ cells, which is adequate only for a 4-kg child [8].

Several approaches have been explored to resolve the issue of inadequacy of HSC numbers in UCB for transplantation. HSC lineages have been derived from human embryonic stem cells [9, 10] and patient-specific HSCs derived from induced pluripotent stem cells (hiPSCs) [11–13]. However, the use of these HSC lineages face clinical challenges as such cells may be fraught with safety concerns such as tumorigenesis and faulty epigenesis [14, 15]. Other approaches include the administration of a second UCB unit to the patient from a different donor or ex vivo expansion of the same patient’s UCB CD34+ cells. Ex vivo expansion of CD34+ cells is more attractive for immunological reasons because cell rejection can be avoided when autologous cells are administered. However, ex vivo expansion protocols must simulate as close as possible in vivo hematopoiesis while at the same time maintaining the stemness properties of the CD34+ expanded cells.

Human mesenchymal stem cells (MSCs) lying within the bone marrow in vivo behave like a scaffold on which HSCs interact and proliferate [5]. To simulate such an environment, autologous MSCs aspirated from the patient’s bone marrow have been grown in vitro to generate a monolayer scaffold on which HSCs have been expanded in numbers with successful results [8, 16, 17]. However, from a practical point of view, the aspiration of patient bone marrow MSCs is an invasive process with the risk of morbidity or infection. Furthermore, the availability of autologous or allogeneic MSC numbers is limited to achieve high fold increases of HSC numbers for transplantation in adults [18–20].

The human umbilical cord (UC) Wharton’s jelly is a rich source of bona fide primitive MSCs (hWJSCs), which have unique stemness properties and increased clinical utility [21]. In previous preliminary studies, we have reported that in the presence of hWJSCs or the conditioned medium in which they are grown (hWJSC-CM), HSCs stick out pseudopodia-like outgrowths and become highly motile. Time-lapse imaging confirmed that the outgrowths were like feet helping them to migrate towards and attach to the surfaces of hWJSCs and undergo proliferation [22]. After 9 days of culture, viability and proliferation assays and FACS analysis showed significant increases in the numbers of CD34^+^ cells compared to controls [22]. Our preliminary studies of the hWJSC secretome reveal a single or combination of several factors in the hWJSC-CM that may be inducing the HSC expansion effects. These factors include members of the interleukin family (IL-6 and IL-8), growth factors (SCF, HGF), glycosaminoglycans (GAGs), hyaluronic acid (HA), cell membrane proteins, cell adhesion molecules, cadherins, and growth factors. Many of these factors have been shown to stimulate hematopoiesis [23–28].

We have pursued these studies further, and in this report, we provide a more in-depth comprehensive evaluation in larger sample numbers of the primitiveness, migration, adhesion, maturation, mitochondrial behavior, and pathway mechanisms of the expansion of human UCB CD34+ cells using hWJSCs and hWJSC-CM as platform support systems. We have also confirmed successful engraftment of such expanded CD34+ cells in in vivo animal experiments to support our in vitro data.

## Material and methods

### Institutional review board approval for use of human cells and animals

Ethical approval with written informed patient consent for the use of human umbilical cords for this study was given by the Institutional Domain Specific Review Board (DSRB), Ministry of Health, Singapore. Commercial human bone marrow mesenchymal stem cells (hBMMSCs) were purchased from Lonza (Basel, Switzerland) and cord blood CD34+ cells from Stem Cell Technologies (Vancouver, Canada). The ethical approval for their use was given by the National University of Singapore, Institutional Review Board (NUS-IRB). Approval for all animal procedures was given by the National University of Singapore Institutional Animal Care and Use Committee (IACUC).

### Cell culture

#### Human Wharton’s jelly stem cells (hWJSCs)

hWJSC lines were derived from human umbilical cords using our previously published protocols [22]. They were cultured in hWJSC medium comprising of 80% DMEM, 20% fetal bovine serum (FBS) (GE Healthcare Life Sciences, UT, USA), 1% non-essential amino acids, 2 mM l-glutamine, 0.1 mM β-mercaptoethanol, 1% insulin-transferrin-selenium, antibiotic/antimycotic mixture (Invitrogen Life Technologies, Carlsbad, CA), and 16 ng/ml basic fibroblast growth factor (Millipore Bioscience, Temecula, CA).

#### Human cord blood CD34+ cells

The cord blood CD34+ cells were thawed at 37 °C and transferred to 15 ml of Iscove’s MDM medium (IMDM) medium (Stem Cell Technologies) containing 10% FBS and centrifuged at 200×*g* for 15 min at room temperature. The supernatant was decanted and the cells used for the experiments.

#### Human bone marrow mesenchymal stem cells (hBMMSCs)

The frozen hBMMSCs (Lonza, Basel, Switzerland) were thawed and cultured in medium consisting of DMEM-high glucose (Invitrogen) supplemented with 10% FBS (GE Healthcare Life Sciences), 1% antibiotic-antimycotic mixture, and 2 mM l-glutamine (Invitrogen).

### Preparation of hWJSC-conditioned medium (hWJSC-CM)

The preparation of hWJSC-CM was carried out as previously described [22]. Briefly, early passages of hWJSCs (P4 to P6) were first cultured in hWJSC medium until 70% confluency. The medium was then sequentially replaced with basal Stemspan SFEM medium (StemCell Technologies) supplemented with 1% antibiotic/antimycotic mixture (Invitrogen). After 24 h, the conditioned hWJSC medium (hWJSC-CM) was collected, filtered through a 0.22-μM filter (Millipore), and stored at − 80 °C until use.

### Characterization of hWJSCs

#### Plastic adherence

hWJSCs grown on the bottom of plastic tissue culture flasks were monitored and imaged with a phase-contrast microscope (Nikon Instruments, Tokyo, Japan).

#### Cell surface markers

Cultured hWJSC monolayers were first disassociated using trypsin (TrypLE™ Express, Thermo Scientific) for 3–5 min at 37 °C in a 5% CO_2_ in air, then centrifuged and washed in phosphate buffered saline (PBS) and blocked with 10% normal goat serum (NGS) (Thermo Scientific) for 10 min at room temperature to prevent non-specific binding following manufacturer’s instruction. The cells were then incubated with mouse monoclonal primary antibodies for a series of CD markers viz., CD14, CD19, CD29, CD34, CD44, CD45, CD73, CD90, CD105, HLA-ABC, and HLA-DR (1:100) (Biolegend, San Diego, CA) for 30 min at 4 °C. This was followed by incubation with Alexa Fluor®488 (1:5000) goat anti-mouse secondary antibody (Thermo Scientific) for 30 min at 4 °C in the dark [29]. The cells were finally washed in PBS (−), re-suspended in 10% NGS, and filtered using a 40-μm nylon strainer (BD) to remove any cell clumps and then analyzed using a CyAn™ ADP Analyzer (Beckman Coulter, Fullerton, CA).

#### Multipotent differentiation of hWJSCs

hWJSCs were seeded (10 × 10^4^ cells/dish) into 6-well tissue culture plates and incubated at 37 °C in a 5% CO_2_ atmosphere for 24 h to allow for cell attachment.

For adipogenic differentiation, the medium was then changed to adipogenic induction medium containing DMEM (Thermo Scientific) supplemented with 10% FBS, 1% penicillin/streptomycin, 0.01 mg/ml insulin (Thermo Scientific), 1 μM dexamethasone (Sigma), 0.5 mM 3-isobutyl-1-methyl-xanthine (IBMX) (Sigma), and 0.2 mM indomethacine (Sigma). The cells were maintained for 21 days in the induction medium, with medium changes twice a week. The cells were then fixed with 4% paraformaldehyde for 10 min, rinsed with PBS, and post-fixed with 60% isopropanol for 5 min. The cells were then stained with Oil Red O for 5 min, rinsed with distilled water, and counter-stained with hematoxylin (Sigma).

For osteogenic differentiation, the hWJSC medium was changed to osteogenic medium containing DMEM medium (Thermo Scientific) supplemented 5% FBS, 0.17 mM l-ascorbic-acid (Sigma, St. Louis, MO), 100 nM dexamethasone, 1% penicillin/streptomycin, and 10 mM β-glycerophosphate (Sigma). The cells were cultured for 21 days with fresh changes of medium twice a week. Osteogenic mineralization was then evaluated by Von Kossa staining. Briefly, the cells were rinsed with PBS and fixed in 4% paraformaldehyde solution (Sigma) for 10 min at room temperature. They were then washed with distilled water and stained in 1% silver nitrate solution (Sigma) under UV light for 60 min. The cells were then counter-stained with 1% nuclear fast red (Sigma) for 5 min.

For chondrogenic differentiation, the hWJSC medium was changed to chondrogenic medium containing DMEM medium (Thermo Scientific) supplemented with 1% penicillin/streptomycin, 1% insulin-transferrin-selenium (ITS), 0.17 mM l-ascorbic-acid, 100 nM dexamethasone, 1 mM sodium pyruvate, 0.35 mM proline, and 10 ng/ml transforming growth factor beta-3 (TGFβ-3) (Sigma). The cells were cultured for 21 days with fresh changes of medium twice weekly. The cells were fixed in 4% paraformaldehyde for 30 min and then stained with 0.5% Alcian Blue (Sigma) for 30 min at room temperature, rinsed with tap water, and then counter-stained with 0.1% Nuclear Fast Red (Sigma) for 5 min. All the stained cells were subsequently visualized and photographed using bright field optics (Nikon Instrument).

### Immunohistochemistry

hWJSCs and hBMMSCs were cultured in a Lab-Tek® Chambered #1.0 Borosilicate coverglass system (Thermo Scientific) until confluence. The cells were then fixed in 4% paraformaldehyde for 10 min, permeabilized with 0.1% Triton-X100 for 10 min, and then washed with PBS. Non-specific blocking was carried out with 10% normal goat serum for 10 min. The cells were incubated with primary antibodies CD29, CD44, CD146, VCAM-1, MMP-2, MMP-9, SDF-1a, ICAM-1, fibronectin, laminin, hyaluronic acid, and collagen type IV (Biolegend, San Diego, USA) at 4 °C overnight. The cells were then incubated with appropriate secondary antibodies (Invitrogen Life Technologies) for 30 min at room temperature in the presence of 1 mg/mL Hoechst 33342 and mounted on to slides with appropriate mounting medium. Photographs were taken using an Olympus FluoView FV1000 laser scanning confocal microscope (Olympus, Japan).

### Experimental design

#### Co-culture of CD34+ cells with hWJSCs or hWJSC-CM

One hundred thousand hWJSCs were seeded per well in 12-well plates (Nalge Nunc International, Rochester, NY, USA). The next day, the hWJSCs were inactivated using mitomycin-C (MMC, 20 μg/mL, NUH Pharmacy, Singapore) and incubated at 37 °C for 2.5 h. The cells were washed and 50,000 CD34^+^ cells were seeded in each well containing the MMC-treated hWJSCs. The two cell types were co-cultured for 7 days at 37 °C in 5% CO_2_ in Stemspan SFEM medium (containing bovine serum albumin, recombinant human insulin, human transferrin, 2-mercaptoethanol, Iscove’s MDM medium (IMDM), and supplements) and CC110 cytokines cocktail (Stem Cell Technologies).

For culture of CD34^+^ cells in hWJSC-CM, 50,000 CD34^+^ cells were seeded in each well of 12 well plates and cultured in 1000 μl of hWJSC-CM for 7 days at 37 °C in 5% CO_2_.

### Experimental analysis

#### Time-lapse and phase-contrast microscopy

The interaction of CD34+ cells with hWJSCs and hWJSC-CM were observed using time-lapse and phase-contrast microscopy during the 7 days of culture. Images of the behavior of the cells were captured using the time-lapse and phase-contrast microscopes (Nikon Instruments, Tokyo, Japan).

#### Cell counts

Aliquots of the CD34+ cells exposed to hWJSCs and hWJSC-CM in all experimental and control groups were collected and stained with 0.4% Trypan Blue (Sigma Chemical Co.) for 1 min at room temperature. The numbers of live cells was counted using a hemocytometer.

#### Surface marker analysis

The cell pellets from experimental and control groups were collected and incubated with conjugated anti-human CD34-APC antibodies (1:10) (Mitenyi Biotec) for 30 min on ice. The cells were also co-stained with anti-human CD133-FITC, CD90-FITC, CD45-FITC, CD49f-FITC (Miltenyi Biotec), and ALDH (Stem Cell Technoglogies). The cells were then washed with PBS, re-suspended in autoMACS running buffer, filtered with a 40-μm strainer, and analyzed with a CyAn™ ADP analyzer (Beckman Coulter, Fullerton, CA, USA).

#### Colony-forming unit (CFU) assay

The cultured CD34+ cells from all experimental and control groups were separated and centrifuged at 300×*g* for 5 min. One thousand cells were then seeded into 35-mm dishes (Nalge Nunc International) containing 0.5 ml of semisolid methylcellulose in Methocult H4435 medium (StemCell Technologies). The 35-mm dishes were incubated at 37 °C in a 5% CO_2_ in air atmosphere for 14 days. Burst forming unit-erythroid (BFU-E), colony-forming unit-granulocyte-erythrocyte-monocyte-megakaryocyte (CFU-GEMM), and colony-forming unit-granulocyte-macrophage (CFU-GM) colonies that were formed after 14 days were counted and classified based on morphology as described by the Atlas of Hematopoietic Colonies from Cord Blood. The CFU colonies were photographed using an inverted phase-contrast microscope (Nikon Instruments, Tokyo, Japan). CFU numbers were then calculated by dividing the number of colonies at day 14 by the number of cells plated and multiplying this value by 10,000 which reflected the colony-forming ability of 10,000 cells.

#### Quantitative real-time polymerase chain reaction (qRT-PCR)

Total RNA from the cultured CD34+ cells in experimental and control groups were extracted using RNeasy Mini kit with gDNA Eliminator Mini Spin Columns (Qiagen, Venlo, the Netherlands). RNA samples were transcribed to cDNA using Tetro cDNA Synthesis kit (Bioline, Eveleigh, NSW, Australia). qRT-PCR was performed with the ABI PRISM 7500 Fast Real-Time PCR System (Applied Biosystems) using Fast SYBR green mastermix (Applied Biosystems) and relative quantification was performed using the comparative CT (2^−ΔΔCT^) method with hGAPDH as the housekeeping gene. The KiCqStart™ primers used were all purchased from Sigma as follows: H_HIF1A_1, H_HK1_1, H_ATP6V1H_1, H_NDUFA_10_1, and H_GAPDH_1.

#### CD34+CXCR4+ analysis

The cell pellets from experimental and control groups were collected and incubated with conjugated anti-human CD34-APC and CXCR4-FITC antibodies (1:10) (Mitenyi Biotec) for 30 min on ice. The cells were washed with PBS, re-suspended in autoMACS running buffer, filtered with a 40-μm strainer, and analyzed with CyAn™ ADP analyzer (Beckman Coulter, Fullerton, CA, USA).

#### N-cadherin analysis

The cell pellet from experimental and control groups were collected and incubated with primary anti-human N-cadherin antibodies (1:100) (Abcam) for 30 min followed by secondary goat anti-mouse IgG (H+L) Alexa Fluor 488 antibodies (1:750) (Invitrogen) for 30 min in the dark. The cells were washed with PBS, re-suspended in 10% NGS, filtered with a 40-μm strainer, and analyzed with CyAn™ ADP analyzer (Beckman Coulter, Fullerton, CA, USA).

#### Transwell migration assay

The cell pellets from experimental and control groups were collected. The cells from each group were then seeded at a density of 1 × 10^5^ cells per well in the upper chamber of a 24-well transwell (8 μm pore size) plate (Corning Costar Corporation, Cambridge, MA, USA) in serum -free medium. Two hundred microliters of serum-containing medium were added to the lower chamber and cell migration from the upper chamber to lower chamber was evaluated after 24 h. The cells that have migrated to the bottom chamber were used for MTS cell viability assay and N-cadherin immunohistochemistry.

#### MTS cell viability assay

For cell viability analysis, MTS assay was performed using Promega CellTiter 96 AQueous One Solution Cell Proliferation Assay kit (Promega Corporation, WI, USA) based on the manufacturer’s instruction. Briefly, 50 μl of MTS reagent was added to the respective CD34+ growth conditions (500 μl) and incubated for about 4 h at 37 °C in a 5% CO_2_ in air atmosphere. Absorbance reading at 490 nm was measured using a spectrophotometer ELISA reader (mQuant; BioTek, Winooski, VT, USA).

#### N-cadherin immunohistochemistry

The migrated cells in the lower chamber were first fixed in 4% paraformaldehyde for 10 min, permeabilized with 0.1% Triton-X100 for 10 min, and then washed with PBS. Non-specific blocking was done with 10% normal goat serum for 10 min. The cells were incubated with primary antibodies N-cadherin (Abcam) at 4 °C overnight. The cells were then incubated with appropriate secondary antibodies (Invitrogen) for 30 min at room temperature in the presence of 1 μg/mL Hoechst 33342, mounted onto slides with appropriate mounting medium. Photographs were taken using an Olympus FluoView FV1000 laser scanning confocal microscope (Olympus, Japan).

#### Mitochondria mass analysis

The cells were washed with PBS and co-cultured with MMC-hWJSCs or hWJSC-CM for 7 days. The cell pellets of CD34+ cells from experimental and control groups were collected and incubated with conjugated anti-human CD34-APC antibodies (1:10) (Mitenyi Biotec) and MitoTracker™-Green dye (Invitrogen) for 30 min on ice. The cells were washed with PBS, re-suspended in autoMACS running buffer, filtered with a 40-μm strainer, and analyzed with a CyAn™ ADP analyzer (Beckman Coulter, Fullerton, CA, USA).

#### CD34+CMXRos analysis

Cell pellets from experimental and control groups were collected and incubated with conjugated anti-human CD34-APC antibodies (1:10) (Mitenyi Biotec) and MitoTracker™ Red CMXRos dye (Invitrogen) for 30 min on ice. The cells were washed with PBS, re-suspended in autoMACS running buffer, filtered with a 40-μm strainer, and analyzed with a CyAn™ ADP analyzer (Beckman Coulter, Fullerton, CA, USA).

#### Mitochondrial superoxide analysis

Cell pellets from experimental and control groups were analyzed for mitochondrial superoxide using the MitoSOX™ Red mitochondrial superoxide indicator kit (Invitrogen). Briefly, the cells were collected and incubated with 100 μL of culture medium containing 0.1 μL of 5 mM MitoSOX™ working solution at 37 °C for 30 min. The cells were then washed, filtered with a 40-μm strainer, and analyzed with a CyAn™ ADP analyzer (Beckman Coulter).

#### Oxidative stress analysis

Cell pellets from experimental and control groups were analyzed for oxidative stress using the CellROX™ Deep Red Reagent (Invitrogen). Briefly, the cells were collected and incubated with 100 μL of culture medium containing 0.2 μL of 2.5 mM CellROX™ working solution at 37 °C for 30 min. The cells were then washed, filtered with a 40-μm strainer, and analyzed with a CyAn™ ADP analyzer (Beckman Coulter).

#### Confocal analysis of co-cultured color-coded CD34+ cells and hWJSCs

MMC-hWJSCs and CD34+ cells were stained with two different color-coded dyes for ease of identification under confocal microscopy. MMC-hWJSCs were incubated with 10 μM of CellTracker™-Red (Invitrogen) for 30 min at 37 °C in a 5% CO_2_ in air atmosphere while the CD34+ cells were stained with 10 μM of MitoTracker™-Green (Invitrogen) at 37 °C for 30 min. The cells for both groups were centrifuged and the media discarded. The cell pellets were washed with PBS before incubation for another 30 min with their own growth media. The CellTracker™-Red-MMC-hWJSCs and MitoTracker™-Green-CD34+ cells were then co-cultured in a Lab-Tek^(R)^ Chambered #1.0 Borosilicate cover glass system for 24 h (Thermo Scientific). The co-culture platform was performed in Stemspan SFEM serum-free medium that contains Iscove’s modified Dulbecco medium with 4500 mg/L of d-glucose and a CC110 cytokines cocktail (Stem Cell Technologies, Singapore Pte Ltd)**.** The pH of the co-culture environment was neither extremely acidic nor alkaline as it readily supported the growth of the CD34+ cells according to the manufacturer’s recommendations. An Olympus FluoView FV1000 laser scanning confocal microscope (Olympus, Japan) was then used to analyze the transfer of mitochondria from CD34+ cells to MMC-hWJSCs.

### In vivo transplantation

Adult 6- to 8-week-old NOD.Cg-Prkdc (scid) Il2rg (tm1Wjll)/SzJ (NSG) (Jackson Laboratory) immunodeficient mice were housed in pathogen-free animal facilities at the NUS Comparative Medicine unit. The mice were first irradiated at 3 Gy 24 h prior. They were then anesthetized with isoflurane and administered intravenously with group 1, CD34+ cells expanded on hWJSCs for 7 days (1 × 10^5^) (experimental), and group 2, uncultured CD34+ cells (1 × 10^5^) (controls). The mice were then sacrificed at 6 weeks post-transplantation and the bone marrow was harvested by flushing the femurs with RPMI medium (Invitrogen). The engraftment of the human cells in the mice was assessed using human FITC-CD45 antibodies (Miltenyi Biotec). The proportion of cells that had > 0.5% labeled with human CD45 was considered positive levels of engraftment and chimerism as recommended by other workers [30]. In the secondary transplantation model, the bone marrow was harvested by flushing the femurs were re-transplanted back to another set of NSG mice. The mice were anesthetized with isoflurane and administered intravenously with group 1, bone marrow cells from primary mice (experimental group), and group 2, bone marrow cells from primary mice (control group). Similarly, the mice were then sacrificed at 6 weeks post-transplantation and the bone marrow was harvested by flushing the femurs, stained with human FITC-CD45 antibodies. The proportion of cells that had > 0.5% labeled with human CD45 was considered positive levels of engraftment and chimerism.

### Statistical analysis

All results were expressed as mean ± SEM and statistical significance between the groups was calculated using the one-way ANOVA or two-tailed Student’s *t* test (SPSS Statistic v 17.0 software package) (SPSS, Inc., IL). The *p* value of < 0.05 was considered statistically significant.

## Results

### Characterization of hWJSCs

**Fig. 1:**
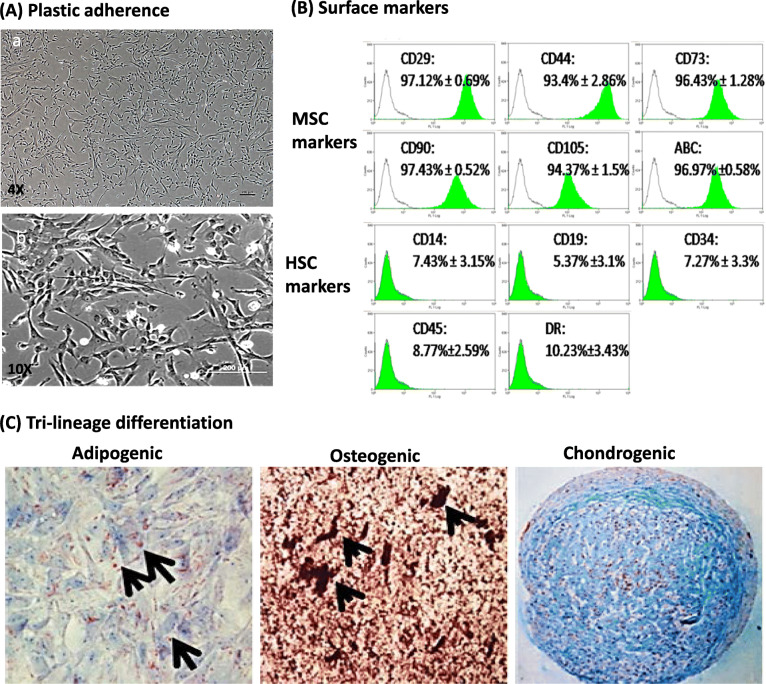
MSC properties of hWJSCs. **A** (a) Low magnification and (b) high magnification of hWJSCs showing plastic adherence in culture and a stellate morphology. **B** hWJSCs express MSC markers such as CD29, CD44, CD73, CD90, CD105, and HLA-ABC but lack expression of hematopoietic markers like CD14, CD19, CD34, CD45, and HLA-DR. **C** hWJSCs differentiate into adipogenic, osteogenic and chondrogenic lineages in vitro

hWJSCs isolated and cultured were plastic adherent and had a stellate fibroblastic-like morphology (Fig. 1A (a, b)). Cell surface marker analysis using flow cytometry showed that hWJSCs were positive for the MSC markers CD105, CD90, CD73, CD29, CD44 and HLA-ABC. The mean percentages ± SEM for each positive CD marker were as follows: CD29, 97.12 ± 0.69%; CD44, 93.4 ± 2.86%; CD73, 96.43 ± 1.28%; CD90, 97.43 ± 0.53%; CD105, 94.37 ± 1.5%; and HLA-ABC, 96.97 ± 0.58%. The hWJSCs were negative for the hematopoietic markers CD14, CD19, CD34, CD45, CD117, and HLA-DR. The mean percentages ± SEM for each negative CD marker were as follows: CD14, 7.43 ± 3.15%; CD19, 5.37 ± 3.1%; CD34, 7.27 ± 3.3%; CD45, 8.77 ± 2.59%; and HLA-DR, 10.23 ± 3.43% (Fig. 1B). The hWJSCs were able to differentiate into adipocytes, osteocytes, and chondrocytes. They showed positive lipid droplet formation with Oil Red O, calcium deposits with von Kossa, and glycosaminoglycans with Alcian blue dye after 21 days (Fig. 1C).

### Immunocytochemistry of hWJSCs for cell adhesion and extracellular matrix markers

**Fig. 2:**
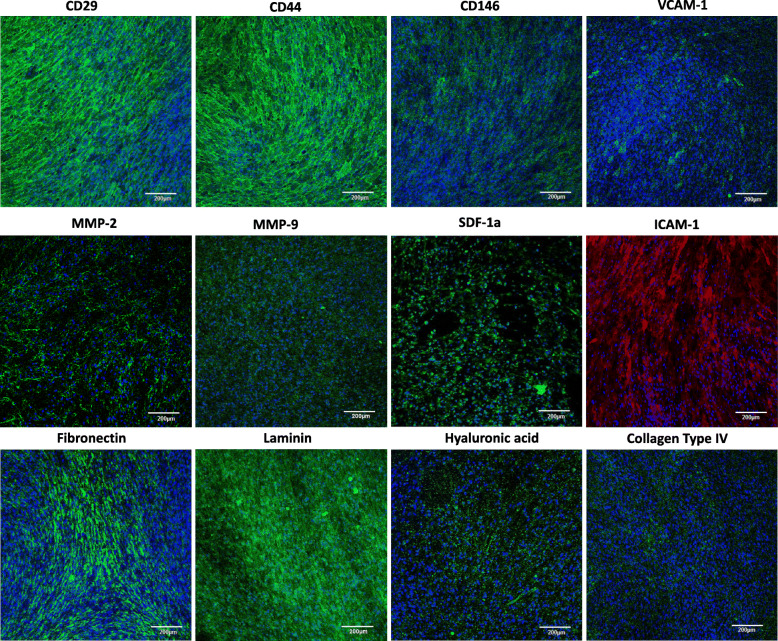
Immunocytochemistry of adhesion and extracellular matrix markers in hWJSCs. hWJSCs stained positive for CD29, CD44, CD146, VCAM-1, MMP-2, MMP-9, SDF-1a, ICAM-1, fibronectin, laminin, hyaluronic acid, and collagen type IV. Magnification × 40; scale bar 100 μM

hWJSCs stained positive for the HSC homing and migration markers such as CD29, CD44, CD146, VCAM-1, MMP-2, MMP-9, SDF-1a, ICAM-1, fibronectin, Laminin, hyaluronic acid, and collagen Type IV (Fig. 2).

### Morphology and fold changes of CD34+ cells expanded with hWJSCs and hWJSC-CM

**Fig. 3:**
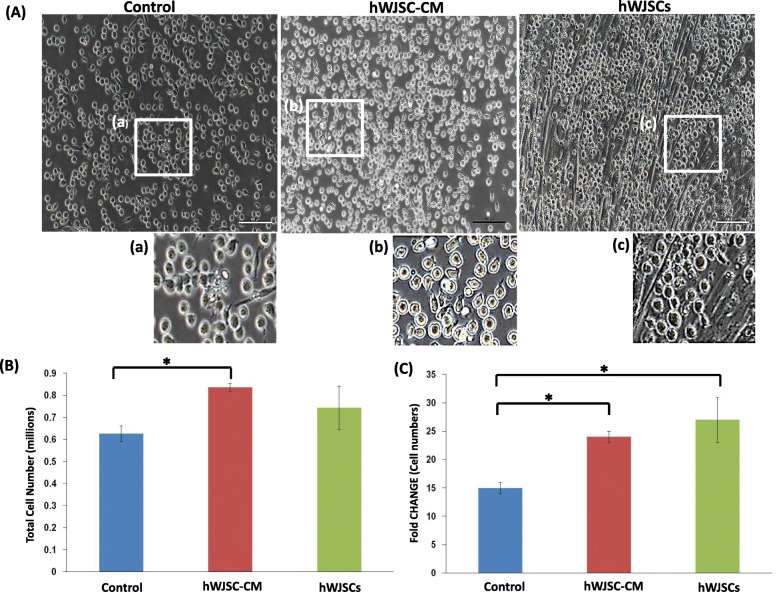
Culture of UCB CD34+ cells with hWJSC-CM and hWJSCs. **A** (a–c) Low-magnification phase-contrast images of CD34+ cells growing in the presence of hWJSCs and hWJSC-CM against controls. Note the larger numbers of CD34+ cells in hWJSCs and hWJSC-CM compared to controls. (d–f) High magnification insets showing pseudopodia-like outgrowths in CD34+ cells expanded in hWJSC-CM (e) and attachment of CD34+ cells to hWJSCs in (f). **B** Note greater total cell numbers of CD34+ cells expanded with hWJSCs and hWJSC-CM as compared to controls (statistically significant between hWJSC-CM and controls). (**C**) Note significant increases in fold changes of CD34+ cell numbers after expansion with hWJSCs and hWJSC-CM compared to controls. All values are expressed as mean ± SEM of 3 biological samples with 3 replicates for each sample. *p < 0.05

The CD34+ cells expanded in the presence of hWJSCs had an elongated morphology and pseudopodia-like outgrowths, and migrated towards and loosely attached to the hWJSCs. They showed similar morphologic changes when they were cultured with hWJSC-CM. This behavior was not observed in the controls as the CD34+ cells continued to show their usual circular morphologies (Fig. 3A (a–f)). There was significantly greater viable CD34+ cell numbers after 7 days of culture with hWJSC-CM. The viable cell counts using trypan blue staining were hWJSCs, 0.74 ± 0.10 × 10^6^; hWJSC-CM, 0.84 ± 0.02 × 10^6^; and controls, 0.63 ± 0.04 × 10^6^ (Fig. 3B). The fold change increases were also significant greater with hWJSCs and hWJSC-CM compared to controls. The fold changes normalized to initial starting number of cells were hWJSCs, 27 ± 4; hWJSC-CM, 24 ± 1; and controls, 15 ± 1 (Fig. 3C).

### Fold changes of cells with primitive HSC markers after expansion with hWJSCs and hWJSC-CM

There were significantly greater numbers of cells with CD34+CD133+, CD34+CD90+, CD34+ALDH+, CD34+CD45+, and CD34+CD49f+ after 7 days of culture in the presence of hWJSCs and hWJSC-CM compared to controls (Table 1). The cell counts for the five primitive markers were [CD34+CD133+: hWJSCs, 0.29 ± 0.07 × 10^6^; hWJSC-CM, 0.26 ± 0.08 × 10^6^; controls, 0.14 ± 0.002 × 10^6^; CD34+CD90+: hWJSCs, 0.44 ± 0.02 × 10^6^; hWJSC-CM, 0.38 ± 0.07 × 10^6^; controls, 0.18 ± 0.01 × 10^6^; CD34ALDH+: hWJSCs, 0.25 ± 0.03 × 10^6^; hWJSC-CM, 0.17 ± 0.03 × 10^6^; controls, 0.06 ± 0.01 × 10^6^; CD34+CD45+: hWJSCs, 0.84 ± 0.006 × 10^6^; hWJSC-CM, 0.93 ± 0.12 × 10^6^; controls, 0.65 ± 0.05 × 10^6^; CD34+CD49f+: hWJSCs, 0.54 ± 0.62 × 10^6^; hWJSC-CM, 0.6 ± 0.07 × 10^6^; controls, 0.39 ± 0.01 × 10^6^].

**Table 1:**
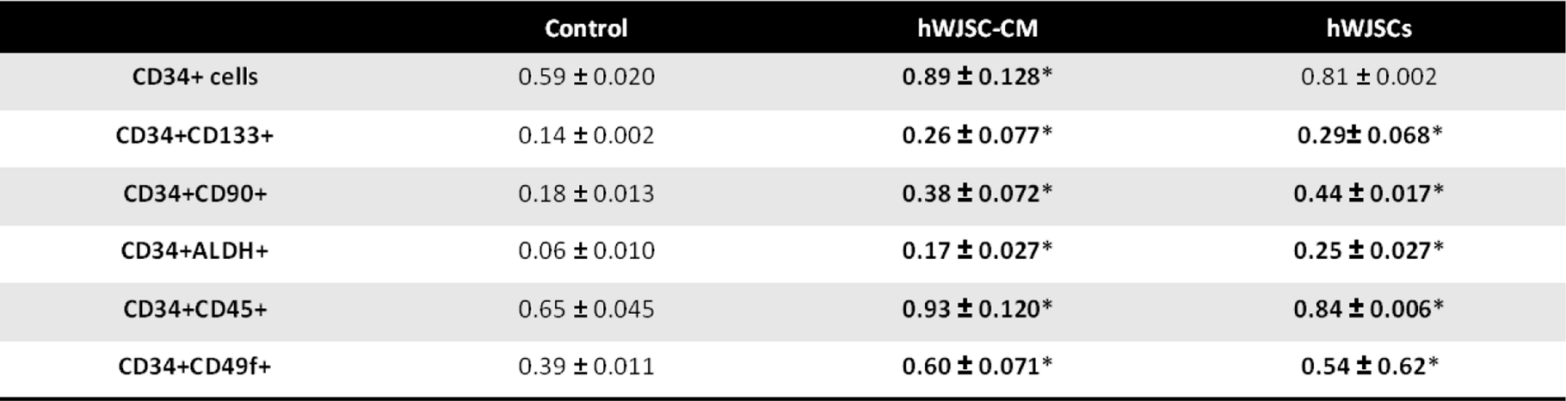
Number (in millions) of cells that is CD34+, CD34+CD133+, CD34+CD90+, CD34+ALDH+, CD34+CD45+, and CD34+CD49f+

There were significant increases in fold change (normalized to initial starting number of cells) of CD34+CD133+, CD34+CD90+, CD34+ALDH+, CD34+CD45+, and CD34+CD49f+ cell numbers after 7 days of culture in the presence of hWJSCs and hWJSC-CM compared to controls (Table 2). The fold change for the five primitive markers (to initial primitive cells seeded) were [CD34+CD133+: hWJSCs, 19 ± 3; hWJSC-CM, 17 ± 3; controls, 9 ± 1; CD34+CD90+: hWJSCs, 2232 ± 294; hWJSC-CM, 1921 ± 67; controls, 1074 ± 58; CD34ALDH+: hWJSCs, 10 ± 1; hWJSC-CM, 11 ± 0 × 10^6^; controls, 4 ± 0; CD34+CD45+: hWJSCs, 26 ± 3; hWJSC-CM, 33 ± 9; controls, 16 ± 2; CD34+CD49f+: hWJSCs, 14 ± 3; hWJSC-CM, 12 ± 1; controls, 7 ± 1].

**Table 2:**
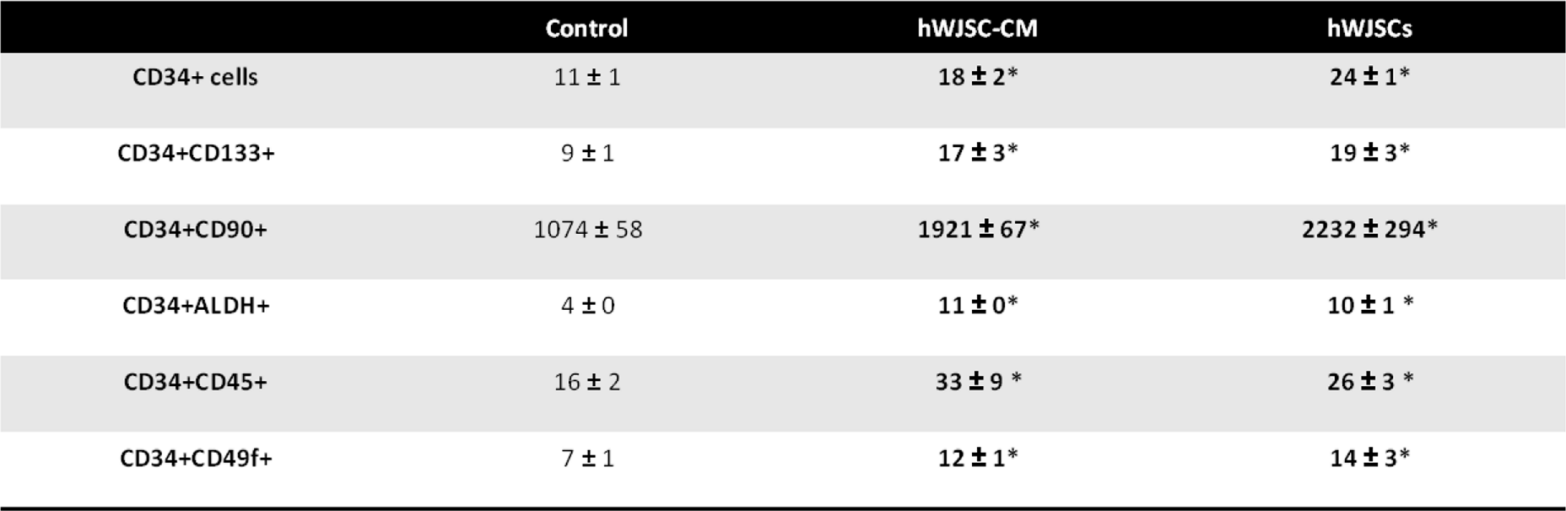
Fold change of cells that is CD34+, CD34+CD133+, CD34+CD90+, CD34+ALDH+, CD34+CD45+, and CD34+CD49f+

^*^p < 0.05

### Colony-forming unit (CFU) numbers and HSC gene expression of CD34+ cells expanded with hWJSCs and hWJSC-CM

**Fig. 4:**
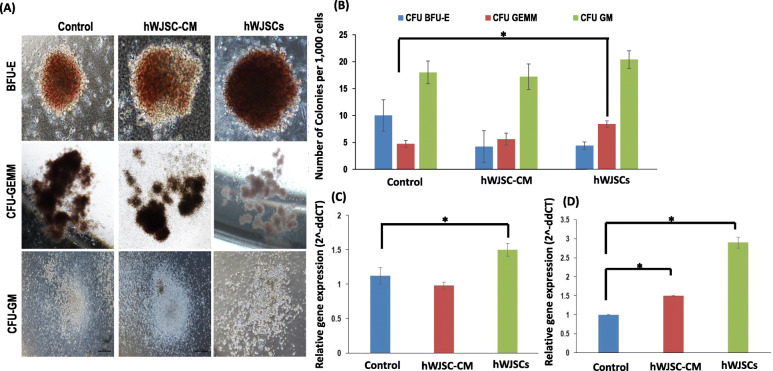
Colony-forming units and primitive HSC gene expression. **a** Representative BFU-E, CFU-GEMM, and CFU-GM colonies of CD34+ cells cultured in control, hWJSC-CM, and hWJSCs for 7 days. **b** Note the significantly greater most primitive CFU-GEMM colonies from CD34+ cells expanded with hWJSCs as compared to controls. **c** Note significantly greater primitive HSC HoxB4 gene expression in CD34+ cells expanded with hWJSCs as compared to controls. **d** Note significantly greater primitive HSC HoxA9 gene expression in CD34+ cells expanded with hWJSCs and hWJSC-CM compared to controls. All values are expressed as mean ± SEM of 3 biological samples with 3 replicates for each sample. *p < 0.05

The CD34+ cells expanded with hWJSCs and hWJSC-CM readily formed CFU in the conventional CFU assay. Colonies with BFU-E, CFU-GEMM, and CFU-GM morphologies from the experimental and control groups were scored (Fig. 4a). There were significantly higher numbers of the most primitive CFU-GEMM colonies from CD34+ cells expanded with hWJSCs compared to hWJSC-CM and controls. The number of CFU-GEMM colonies per 1000 cells were hWJSCs, 8 ± 1; hWJSC-CM, 6 ± 1; and control, 5 ± 1 (Fig. 4b).

There were no significant differences in the number of the lesser primitive BFU-E and CFU-GM colonies between the hWJSCs, hWJSC-CM, and control groups. The number of BFU-E colonies per 1000 cells were hWJSCs, 4 ± 1; hWJSC-CM, 4 ± 3; and control, 10 ± 3. The number of CFU-GM colonies per 1000 cells were hWJSCs, 20 ± 2; hWJSC-CM, 17 ± 2; and control, 18 ± 2.

There was significantly greater primitive HSC HoxB4 gene expression in CD34+ cells expanded with hWJSCs as compared to hWJSC-CM and controls (Fig. 4c). The relative quantification (2^−ΔΔCT^) of HoxB4 normalized to housekeeping and controls were hWJSCs, 1.5 ± 0.09; hWJSC-CM, 0.98 ± 0.05; and control, 1.12 ± 0.12. Also, the levels of HoxA9 gene expression in CD34+ cells expanded with hWJSC-CM and hWJSCs were significantly greater than controls (Fig. 4d). The relative quantification (2^−ΔΔCT^) of HoxA9 normalized to housekeeping and control were hWJSCs, 2.90 ± 0.15; hWJSC-CM, 1.5 ± 0.01; and controls, 0.94 ± 0.

### Cell adhesion markers and migration of CD34+ cells expanded with hWJSCs and hWJSC-CM

**Fig. 5:**
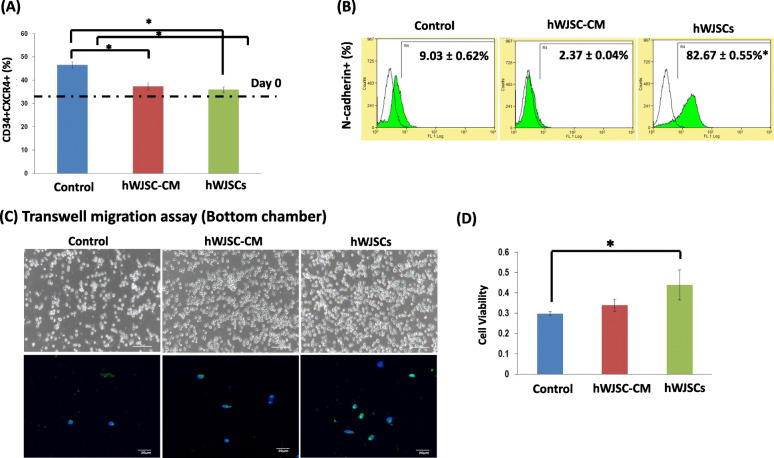
Cell adhesion markers and migration of UCB CD34+ cells expanded with hWJSCs and hWJSC-CM. **A** Note the percentage of the most primitive CD34+CXCR4+ cells in the hWJSCs and hWJSC-CM-expanded groups was closest to the initial seeding cell numbers on day 0 (dotted line) compared to the controls. **B** Note significantly greater percentage of N-cadherin+ cells after expansion of CD34+ cells with hWJSCs as compared to controls. **C** (a–c) Phase-contrast images of the bottom chamber of transwell migration assays. Note increased number of CD34+ cells that have migrated from the top to the bottom chamber when cultured in the presence of hWJSCs and hWJSC-CM compared to controls. (d–f) There was an increased number of N-cadherin positive cells among the migrated cells expanded with hWJSCs and hWJSC-CM compared to controls. **D** Note significantly greater viable CD34+ cell numbers (MTS assay) lying in the bottom chamber of transwell migration chambers after expansion with hWJSCs as compared to controls. All values are expressed as mean ± SEM of 3 biological samples with 3 replicates for each sample. *p < 0.05

The percentage of the most primitive unmanipulated CD34+CXCR4+ cells in the hWJSCs and hWJSC-CM expanded groups was closest to the initial seeding cell numbers on day 0 (dotted line) compared to the controls (Fig. 5A). The percentages of CD34+CXCR4+ cells were hWJSCs, 35.91 ± 1.18%; hWJSC-CM, 37.33 ± 1.44%; and controls, 46.53 ± 1.43% (Fig. 5A).

There were significant increases in the percentage of N-cadherin+ cells after 7 days of culture with hWJSCs compared to controls. The percentages of N-cadherin+ cells were hWJSCs, 82.67 ± 0.55%; hWJSC-CM, 2.37 ± 0.04%; and controls, 9.03 ± 0.62% (Fig. 5B).

In the transwell migration assays, there were more cells that were expanded with hWJSCs and hWJSC-CM for 7 days migrating to the bottom chamber as compared to controls. Most of these cells in the bottom chamber in the hWJSCs and hWJSC-CM groups were N-cadherin positive compared to controls (Fig. 5C). Also, the MTS viability assay showed that the number of viable cells in the bottom chamber were significant greater in the hWJSC-expanded group compared to hWJSC-CM and controls. The cell viability percentages were hWJSCs, 0.44 ± 0.07; hWJSC-CM, 0.34 ± 0.03; and control, 0.30 ± 0.0 (Fig. 5D).

### Mitochondrial behavior of CD34+ cells expanded with hWJSCs and hWJSC-CM

**Fig. 6:**
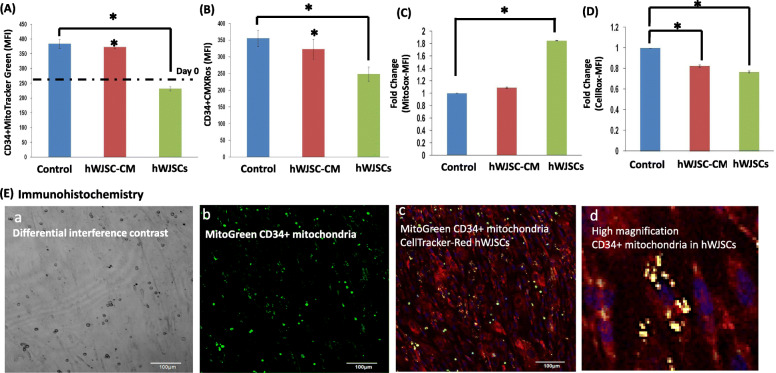
Mitochondrial behavior of UCB CD34+ cells expanded with hWJSCs and hWJSC-CM. **A** Note that there were greater increases in the CD34+MitoTracker-Green staining (Mean fluorescence index; MFI) cells expanded in hWJSC-CM after 7 days compared to their numbers at day 0. **B** Note significantly lower mitochondrial membrane potential in CD34+CMXRos+ cells expanded with hWJSCs as compared to controls. **C** Note significantly increased mitochondrial superoxide staining in CD34+ cells expanded with hWJSCs as compared to controls. **D** Note significantly lower oxidative stress (CellRox staining) in CD34+ cells expanded with hWJSCs and hWJSC-CM as compared to controls. All values are expressed as mean ± SEM of 3 biological samples with 3 replicates for each sample. Asterisk (*): *p* < 0.05. **E** (a–d) Confocal immunohistochemistry images of Mitotracker-Green CD34+ cells interacting with CellTracker-Red hWJSCs. **a** Low-magnification confocal image of co-culture of the two cell types under differential interference contrast (DIC) optics. **b** Low-magnification confocal image of MitoGreen CD34+ mitochondria. **c** Low-magnification confocal image of MitoGreen CD34+ cells co-cultured with CellTracker-Red-hWJSCs. **d** High magnification of Mitotracker-Green CD34+ cells co-cultured with CellTracker-Red hWJSCs. Note MitoTracker-Green CD34+ mitochondria lying inside the cytoplasm of CellTracker-Red hWJSCs (yellow dots)

There were greater increases in the CD34+MitoTracker-Green staining (mean fluorescence index; MFI) cells expanded in hWJSC-CM after 7 days compared to their numbers at day 0. The CD34+MitoTracker-Green (MFI) values were hWJSCs: 231.6 ± 6.96; hWJSC-CM: 373.3 ± 6.4 and Controls: 384.0 ± 15.1 (Fig. 6A). There were significant decreases in the CD34+CMXRos staining (mitochondria membrane potential) after 7 days of culture with hWJSCs compared to hWJSC-CM and controls. The CD34+CMXRos staining (MFI) values were hWJSCs, 248.8 ± 21.3; hWJSC-CM, 322.97 ± 29.88; and controls, 355.8 ± 24.4 (Fig. 6B). There were significant increases in mitochondrial superoxide (MitoSox) staining (mitochondria stress) after 7 days of culture with hWJSCs compared to hWJSC-CM and controls. The MitoSox staining (MFI) values were hWJSCs, 1.85 ± 0.001; hWJSC-CM, 1.09 ± 0.01; and controls, 1.0 ± 0 (Fig. 6C). There were significant decreases in the oxidative stress (CellROX) staining after 7 days of culture in the hWJSCs and hWJSC-CM groups compared to controls. The fold changes in CellROX staining (MFI) were hWJSCs, 0.77 ± 0.01; hWJSC-CM, 0.82 ± 0.01; and controls, 1.00 ± 0 (Fig. 6D). Confocal microscopic images showed MitoTracker-Green-tagged mitochondria from the CD34+ cells residing inside the MMC-hWJSC-Red-tagged cells during the expansion process (Fig. 6E (a–d)).

### Glycolysis and oxidative phosphorylation-related gene expression of CD34+ cells expanded with hWJSCs and hWJSC-CM

There was significantly greater glycolytic pathway-related HIF-1a and HK-1 gene expression in CD34+ expanded with hWJSCs as compared to hWJSC-CM and controls (Fig. 6A, B). The relative quantification (2^−ΔΔCT^) of HIF-1a normalized to housekeeping and controls were hWJSCs, 1.20 ± 0.53; hWJSC-CM, 0.35 ± 0.08; and controls, 0.89 ± 0.12. The relative quantifications (2^−ΔΔCT^) of HK-1 normalized to housekeeping and controls were hWJSCs, 1.19 ± 0.35; hWJSC-CM, 0.72 ± 0.12; and controls, 1.00 ± 0.

There was significantly lower oxidative phosphorylation pathway-related ATP6VIH and NDUFA10 gene expression in CD34+ cells expanded with hWJSCs and hWJSC-CM compared to controls (Fig. 6C, D). The relative quantifications (2^−ΔΔCT^) of ATP6VIH normalized to housekeeping and control were hWJSCs, 0.39 ± 0.11; hWJSC-CM, 0.31 ± 0.04; and controls, 0.88 ± 0.04 and for NDUFA10 were hWJSCs, 0.87 ± 0.31; hWJSC-CM, 0.44 ± 0.06; and controls, 0.87 ± 0.06.

### In vivo transplantation model for cord blood CD34+ cells expanded on hWJSCs

**Fig. 7:**
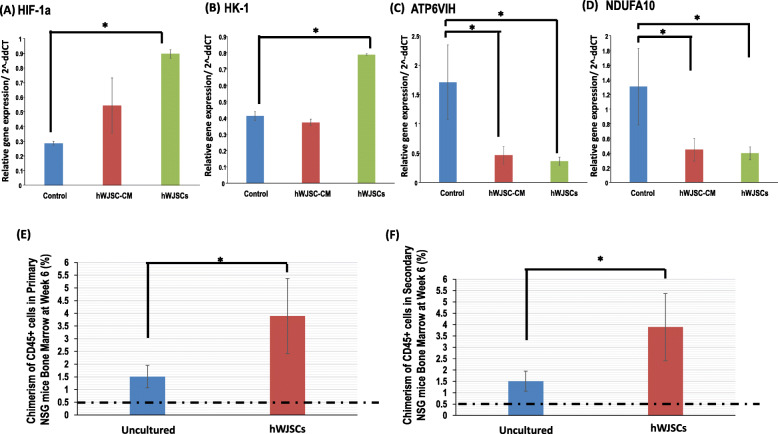
Glycolysis (HIF-1a and HK-1) and oxidative phosphorylation (ATP6VIH and NDUFA10) gene expression of UCB CD34+ cells expanded with hWJSCs and hWJSC-CM. **a** Note significantly greater HIF-1a and **b** HK-1 gene expression in CD34+ cells expanded with hWJSCs as compared to controls. **c** Note significantly lower ATP6VIH and **d** NDUFA10 gene expression in cells expanded with hWJSC-CM and hWJSCs as compared to controls. All values are expressed as mean ± SEM of 3 biological samples with 3 replicates for each sample. *p < 0.05. In vivo transplantation of hWJSC-expanded CD34+ cells into NSG mice. **e** Note significantly greater CD45+ chimerism in the bone marrow of primary NSG mice at 6 weeks post-transplantation with CD34+ cells expanded with hWJSCs as compared to uncultured UCB CD34+ cells. **f** Note significantly greater CD45+ chimerism in the bone marrow of secondary NSG mice at 6 weeks post-transplantation with cells isolated from bone marrow of primary NSG mice and expanded with hWJSCs as compared to uncultured UCB CD34+ cells. All values are expressed as mean ± SEM of 4 biological samples. *p < 0.05

There were significantly greater percentages of human CD45+ chimerism in the primary NSG mice when transplanted with CD34+ cells (expanded with hWJSCs for 7 days) as compared to day-0 CD34+ cells (Fig. 6E). The percentage chimerism of human CD45+ cells in the bone marrow of the primary mice at week 6 post-transplantation were as follows: day-7-hWJSC-expanded, 3.89 ± 1.48%, and day 0, 1.5 ± 0.44%. When the bone marrow cells from the primary NSG mice were re-transplanted to secondary mice, there were significantly greater percentages of CD45+ chimerism in the secondary mice for the 7-day CD34+ cells expanded on hWJSCs as compared to day-0 CD34+ cells (Fig. 6F). The percentage chimerism of human CD45+ in the bone marrow of the secondary NSG mice at week 6 post-transplantation were as follows: day-7-hWJSC-expanded, 1.15 ± 0.14%, and day-0, 0.67 ± 0.09% (Fig. 7).

## Discussion

Hematopoietic stem cells from the umbilical cord blood (UCB) could differentiate into all mature blood lineages and have been used for the treatment of blood disorders clinically. However, the low numbers of HSCs present in the UCB and their low self-renewal in vitro are the major hurdles that limit their use. One of the therapeutic strategies to help increase UCB-HSC numbers is to develop reliable ex vivo expansion protocols [5].

Some of the approaches that have been attempted are the use of stromal cells as supportive monolayers [31], genetically modified cytokine-releasing cells [32], human bone marrow mesenchymal stem cells (hBMMSCs) [33], and immortalized hBMMSCs [34] for the ex vivo expansion of HSCs. The hypothesis with respect to mechanism of action was that these feeder cells are able to produce a sustained release of molecules that promote HSC maintenance and proliferation [8]. Autologous or allogeneic hBMMSCs thus far have been the cell of choice for stromal support of HSCs since hBMMSCs are the normal resident MSCs in the bone marrow that serve as natural scaffold dispensing molecules for the expansion and maintenance of HSCs in vivo. On top of the risks of morbidity and infection to donors during bone marrow aspiration, and the limited numbers of MSCs available, hBMMSCs were also shown to transform into tumor-associated fibroblasts in the tumor microenvironment [35]. As such, there will be limitations for the use of hBMMSCs as a platform for HSC expansion clinically.

hWJSCs isolated from the Wharton’s jelly compartment of discarded umbilical cords is an attractive alternative source for allogeneic or autologous MSC stromal support for HSC expansion [22]. They share common developmental origins as hBMMSCs [36], satisfy the criteria for bona fide MSCs as recommended by the International Society of Cytotherapy (ISCT) [37] and are primitive possessing their unique secretome. They are also hypo-immunogenic, non-tumorigenic, and do not induce toxicity in animal models and in human clinical trials thereby enabling their safe usage clinically [38–42]. Their harvest is non-invasive; they are available in abundance from discarded umbilical cords and have short population doubling times retaining their stemness properties for over fifty passages [3]. We have shown previously that nearly 4–5 × 10^6^ hWJSCs/cm of human umbilical cord can be consistently isolated [43, 44]. They are thus an excellent source of allogeneic “off-the-shelf” cells that can be manufactured under good manufacturing conditions (GMP) as a clinically viable platform for ex vivo expansion of UCB-HSCs.

The time-lapse microscopy and phase-contrast images of this study showed that CD34+ HSCs undergo a very unique behavior in the presence of hWJSCs or hWJSC-CM putting out pseudopodia-like structures which they use for active motility and migration towards the surface of hWJSCs where they attach and proliferate. Since this behavior was observed in both hWJSCs and hWJSC-CM groups, it is clear that certain molecules released by the hWJSC secretome initiate and maintain this behavior. Direct contact of HSCs with MSCs has been shown to affect migratory behavior and gene expression profiles of CD133+ HSCs during ex vivo expansion [17]. The results of this study showed that the expansion of UCB CD34+ cells in the presence of hWJSCs and hWJSC-CM significantly increased the numbers and fold changes of primitive HSC CD34+CD133+, CD34+CD90+, CD34+CD45+, and CD34+ALDH+ cells. These markers are the more commonly recognized primitive stemness surface markers for HSCs. Likewise, during the CD34+ cell expansion processes, the primitive HSC gene-related expression such as HoxB4 and HoxA9 was also significantly increased. These results suggest that the normal maturation of HSCs is in progress using this expansion platform.

The stromal cell monolayers function as specific stem cell niches for HSC migration and proliferation. While multiplying in numbers in the liquid medium milieu of the conditioned medium, we also noticed that the CD34+ cells also attach to the surface of hWJSCs and actively proliferate in numbers. It has been hypothesized that the surface of MSCs was the predominant site that HSCs proliferate [16]. It has also been reported that these natural surface niches attract and anchor CD34+ HSCs, and the process involves N-cadherins, CD44, VCAM-1, Jagged-1, and other integrins [45]. The role of these adhesion proteins is to mediate HSC anchorage to the niche and maintain the HSC stemness. The immunocytochemistry staining in the results of the present study indicates that hWJSCs express adhesion and extracellular matrix molecules such as CD29, CD44, CD146, VCAM-1, MMP-2, MMP-9, SDF-1a, ICAM-1, fibronectin, laminin, hyaluronic acid, and collagen type IV that are required for HSC migration and homing. In fact, our results showed that the CD34+ cells expanding on hWJSCs had increased migration ability. Our results also showed that UCB CD34+ cells had increased expression of N-cadherins after expansion with hWJSCs. It has been previously shown that MSCs from human bone marrow and cord blood had better hematopoietic supporting function during stromal support when compared to adipose tissue-derived MSCs due to a higher propensity for UCB CD34+ cells to anchor to the stromal cell layers via N-cadherins [46]. The increased N-cadherin expression in the CD34+ cells expanded on hWJSCs in the present study demonstrates better adherence and maintenance of their primitiveness.

The expression of N-cadherins on CD34+ HSCs has been previously shown to support the maintenance of long-term culture initiating cells (LTC-IC), which is consistent with the results of this study [45]. We have observed that CD34+ cells expanded on hWJSCs had increased ability to form CFU-GEMM and LTC-IC (unpublished), which suggest their primitiveness. Furthermore, the in vivo animal results in the present study that showed high levels of human CD45+ chimerism in the bone marrow of primary NSG mice transplanted with CD34+ cells expanded with hWJSCs confirmed optimal hematopoietic engraftment. The long-term repopulating abilities were further confirmed by increased human CD45+ chimerism in the bone marrow of secondary transplanted NSG mice.

Studies have recently shown that the change of stemness identity and functions of early HSC commitment involved a profound change in in the metabolism of HSCs [47, 48]. Long-term HSCs that are mostly non-dividing tend to use the anaerobic glycolysis pathway to produce energy, while the progenitor cell types that have lesser self-renewal abilities produces energy using mitochondria via the oxidative phosphorylation pathway [49, 50]. The key difference in the energy metabolism pathways plays an important role in maintaining their long-term repopulating capabilities in vivo. Our results showed that UCB CD34+ cells expanded with hWJSCs and hWJSC-CM had lower expression of genes related to oxidative phosphorylation such as ATP6VIH and NDUFA10. Also, the present study showed that CD34+ cells expanded with hWJSCs had higher expression of HIF-1a and HK-1. Interestingly, HIF-1a is the master gene regulator for glycolysis [47]. Our results demonstrate for the first time a link between the hWJSC expansions of UCB CD34+ cells to metabolic pathways. The low mitochondrial respiration in the hWJSCs may be a protection against cellular damage by reactive oxygen species (ROS) from active mitochondria [48, 50]. In fact, we observed that UCB CD34+ cells expanded with hWJSCs and hWJSC-CM showed lower levels of reactive oxygen species. It has been shown previously that low ROS select for primitive HSC [51]. It was also reported in an in vitro tracking expansion culture that the actively self-renewing HSCs had low mitochondrial mass and activities while differentiating cells had higher mitochondrial mass and activities [47]. In our study, we have also observed that the UCB CD34+ cells expanded on hWJSCs had lower mitochondrial mass and lower mitochondria membrane potential which correlates with mitochondrial behavior previously reported [47]. Likewise, mitochondrial superoxide was higher after expansion of CD34+ cells in the presence of hWJSCs.

It was shown previously that primitive Tie2+ HSCs keep their primitiveness through mitochondrial mitophagy [52]. Mitochondrial clearance was also observed in the present study in CD34+ cells expanded with hWJSCs. The green-tagged mitochondria from the CD34+ cells were found within the cytoplasm of red-tagged hWJSCs showing transfer from cell to cell during the co-culture process. This transfer may be occurring via tunneling nanotubes produced by the hWJSCs, which have been shown to be involved in intercellular exchange of components between neighboring cells including mitochondrial transfer. It has previously been shown that hWJSCs formed nanotubes with neighboring cells and transfer mitochondria between them [53]. Studies have shown that transfer of subcellular material like mitochondria in nanotubes between MSCs and injured tissues could be a mechanism that MSCs use to repair and treat diseases [53].

Transfer of cellular cargo via tunneling nanotubes (TNTs) between MSCs and neighboring cancer cells or other injured cells was shown to be unidirectional or bi-directional [54]. In most cases however, MSCs were shown to transfer their mitochondrial cargo through a TNT-mediated process to target cells [55]. Conversely, the reverse process has been reported, where when vascular smooth muscle cells (VSMC) were co-cultured with MSCs, the TNT-mediated mitochondrial trafficking resulted in the transfer of the VSMC mitochondria to the MSCs [56]. Based on our time-lapse and confocal microscopy studies of the behavior of the two types of color-tagged cells, we observed that similar to VSMCs the transfer of mitochondria was unidirectional and was taking place from the CD34+ cells to the hWJSC/MSCs. The formation of tunneling nanotubes (TNTs) when tested in two dimensional in vitro cultures was observed to be controlled by several factors including serum and glucose concentrations [55]. Low-serum and high-glucose concentrations in vitro were found to stimulate TNT formation and mitochondrial trafficking [57, 58]. Hence, the glucose levels in the Stemspan SFEM serum-free medium of the present study would have adequately stimulated TNT formation in the hWJSCs to allow mitochondrial transfer.

The CC110 cytokine cocktail contains a battery of cytokines including common hematopoietic stem cell growth factors such as SCF, Flt3-L, and TPO. Such cytokines are known to modulate TNT formation [54, 55, 59]. Additionally, we have shown previously that the top 10 most highly differentially overexpressed genes in hWJSCs compared with human bone marrow MSCs (hBMMSCs) were CXCL8 (IL8), CXCL1, IL1B, DSG2, ST6GALNAC5, PCDH10, CXCL6, KRTAP7-1, DSC3 (Desmocollin 3), and NEFL [60]. These genes upregulated in hWJSCs play key roles in processes contributing to the success of hWJSCs as a therapeutic source of stem cells including immunomodulation, angiogenesis, wound healing, apoptosis, antitumor activity, and chemotaxis. The production of cytokines by the overexpression of these genes would have also assisted in TNT formation and the mitochondrial trafficking.

The hWJSC/MSCs first lay down an extracellular matrix that secretes molecules to make the CD34 + cells active, increase their mitotic activity, and attract them to adhere to the matrix and stimulate their proliferation. We have shown previously in time-lapse confocal microscopy observations that when CD34+ cells are exposed to hWJSCs, they put out pseudopodia-like structures, become very motile and migrate towards the hWJSCs, adhere to them, and undergo proliferation. This was confirmed with scanning electron microscopy studies as well [22]. Other groups have also described the chemo-haptotaxis of CD34+ cells towards stromal MSCs, hWJSCs, or its extracellular matrix (ECM) [61, 62]. More recently, it was shown that a decellularized Wharton jelly stem cell matrix used as a biomimetic scaffold enhanced ex vivo hematopoietic stem cell expansion in culture. The authors showed that the decellularized Wharton jelly stem cell matrix induced CXCR4 expression molecularly and phenotypically in UCB CD34+ cells which enhanced their transmigration capability [63]. In the present study too, time-lapse confocal microscopy showed the migration of CD34+ cells towards the hWJSC monolayer with their adherence and subsequent multiplication. The present study also confirmed that hWJSCs expressed high levels of VCAM-1 which has been shown to promote primitive HSC transmigration [64].

During adhesion to the hWJSC/MSCs, the primitive CD34+ cells shed their excessive mitochondria that are transferred to hWJSC/MSC to help them remain primitive and maintain their stemness properties during proliferation. This may be a unique peculiarity of hWJSCs. We have not seen any proof of cargo transfer in the reverse direction from hWJSCs to CD34+ cells in our time-lapse and confocal microscopy observations. This is the first report showing the unidirectional flow of mitochondrial cargo from a primitive CD34+ cell to a primitive MSC that has acquired different and unique properties compared to conventional MSCs because of its residence in the human umbilical cord which is protected from the external insults of the environment during pregnancy.

In the human body, MSCs in the bone marrow act like a scaffold or matrix dispensing molecules for the proliferation of neighboring hematopoietic stem cells (HSCs) or CD34+ cells. Our hWJSC monolayers mimic this phenomenon by providing such an extracellular matrix or scaffold for migration, attachment, and proliferation of CD34+ cells. The increased mitotic activity, migration, and adherence of the CD34+ cells to the hWJSC matrix are via the increased expression of N-cadherins by the hWJSCs.

Additionally, the presence of the hWJSC monolayer causes the clearance of mitochondria from the expanded CD34+ cells, keeping them primitive. The mitochondrial clearance mechanism enables the CD34+ cells to use the glycolytic energy metabolism pathway and protect the cells from cellular damage from reactive oxygen species (ROS) due to active mitochondria [48, 50].

This paper has demonstrated that allogeneic hWJSCs can be harvested and provide a useful source of stromal support for the ex vivo expansion of UCB CD34+ cells and helping to keep them primitive. Moreover, we have previously demonstrated that hWJSCs can be harvested and used as stromal support for the expansion of UCB CD34+ cells within 24 h [22]. Thus, hWJSCs could be isolated, stored, and cultured using cGMP-compliant protocols that will greatly promote their use clinically [65, 66]. As such, the hWJSC expansion platform described in this study could be used clinically in cord blood transplantation settings as both hWJSCs and the cord blood CD34+ cells culture reagents could be made cGMP-compliant. We have shown in previous publications that hWJSCs are safe as they do not undergo malignant transformation in long-term culture and do not form tumor-associated fibroblasts (TAFs) unlike bone marrow MSCs (hBMMSCs) [34, 39]. The feasibility to freeze and store allogeneic hWJSCs has immense benefits as they could then be thawed and used for UCB-HSC expansion if the numbers were inadequate. The use of allogeneic hWJSCs as an ex vivo support system for cord blood HSCs would also help to improve HSC transplantation engraftment efficiencies greatly, as this approach will avoid the need for discarding precious HSC samples with low cell numbers as is practiced by cord blood banks today [67].

In conclusion, the greatest limitation in the use of CD34+ cells (HSCs) for successful transplantation and treatment of malignant hematopoietic diseases are the low cell numbers available. The novelty and added advantages of the platform technology in our studies is that hWJSCs through the release of their extracellular matrix (ECM) proteins are able to provide stromal support for the attraction, adherence, and increased proliferation several-fold of CD34+ cells that remain primitive and generate all the matured hematopoietic lineages. hWJSCs themselves possess high proliferation rates and can be harvested in large numbers from discarded umbilical cords, and GMP-compliant allogeneic hWJSC monolayers are easy to prepare with consistent results for routine use in cord blood bank settings to expand several-fold primitive CD34+ cells for use in patients.

## Data Availability

Supporting data can be obtained from the corresponding author.

## Abbreviations

ATP6VIH: ATPase, H+ transporting, lysosomal 50/57 kDa, V1 subunit H
BFU-E: Burst forming unit
CFU: Colony-forming unit
CFU-GEMM: Colony-forming unit-granulocyte-erythrocyte-monocyte-megakaryocyte
CFU-GM: Colony-forming unit-granulocyte-macrophage
DMEM: Dulbecco’s modified Eagle’s medium
ECM: Extracellular matrix
FBS: Fetal bovine serum
GAGs: Glycosaminoglycans
HA: Hyaluronic acid
HIF-1a: Hypoxia-inducible factor-1 alpha
HK-1: Hexokinase-1
HSCs: Hematopoietic stem cells
HPCs: Hematopoietic progenitor cells
hiPSCs: Human induced pluripotent stem cells
hBMMSCs: Human bone marrow mesenchymal stem cells
hWJSCs: Human Wharton’s jelly stem cells
hWJSC-CM: Human Wharton’s jelly stem cells-conditioned medium
ICAM-1: Intercellular adhesion molecule 1
IL-6: Interleukin-6
IL-8: Interleukin-8
IMDM: Iscove’s modified Eagle’s medium
ITS: Insulin-transferrin-selenium
MMC: Mitomycin-C
MMP-2: Matrix metallopeptidase 2
MMP-9: Matrix metallopeptidase 9
MSCs: Mesenchymal stem cells
NDUFA10: NADH:Ubiquinone Oxidoreductase Subunit A10
NGS: Normal goat serum
NSG: NOD.Cg-Prkdc (scid) Il2rg(tm1Wjll)/SzJ
qRT-PCR: Quantitative real-time polymerase chain reaction
ROS: Reactive oxygen species
SDF-1a: Stem cell derived factor-1
SCF: Stem cell factor
UC: Umbilical cord
UCB: Umbilical cord blood
VCAM-1: Vascular cell adhesion molecule 1
